# Single‐cell analyses reveal impaired type B spermatogonia differentiation and meiotic entry in C‐Nap1‐null testes

**DOI:** 10.1002/qub2.71

**Published:** 2024-11-26

**Authors:** Junlin Li, Liheng Yang, Liansheng Li, Min Li, Juntao Gao, Li Yuan

**Affiliations:** ^1^ Medical School University of Chinese Academy of Sciences Beijing China; ^2^ Zanvyl Krieger School of Arts and Sciences Johns Hopkins University Baltimore Maryland USA; ^3^ Viterbi School of Engineering University of Southern California Los Angeles California USA; ^4^ Molecular Biology Laboratory Guang’anmen Hospital China Academy of Chinese Medical Sciences Beijing China; ^5^ Institute for TCM‐X Beijing China; ^6^ MOE Key Laboratory of Bioinformatics Beijing China; ^7^ Bioinformatics Division Department of Automation BNRist Beijing China; ^8^ Center for Synthetic & Systems Biology Tsinghua University Beijing China

**Keywords:** C‐Nap1, mouse testis, single‐cell RNA sequencing, spermatogenesis

## Abstract

Sperm development is critical for male reproductive capability; any disruption during the process of spermatogenesis will result in male infertility. In this research, we used the C‐Nap1 encoded by the gene of *Cep250* knockout mouse line as the model to evaluate the impact of absent C‐Nap1 on spermatogenesis. To investigate the interaction between C‐Nap1 and spermatogenesis, we utilized single‐cell RNA sequencing to analyze 10,332 *C‐Nap1* ^*+/+*^ and 13,308 *C‐Nap1* ^*−/−*^ testicular cells. We identified five main cell types within seminiferous tubules, including spermatogonia, Sertoli cells, spermatogonia stem cells, Leydig cells, and spermatocytes. We found a critical reduction in testicular spermatogonia and spermatocytes in C‐Nap1‐null testes, compared to its *C‐Nap1* ^*+/+*^ controls. By combining uniform manifold approximation and projection clustering and psedotime ordering, we distinguished five spermatogonial stages/subtypes, demonstrating that type B spermatogonia differentiation and meiotic initiation are impaired during C‐Nap1‐null spermatogenesis. Following gene ontology enrichment analysis, meiosis‐specific genes downregulated in the *C‐Nap1* ^*−/−*^ testicular cells were further verified by reverse transcription polymerase chain reaction (RT‐PCR). Based on the differential gene expression, certain downregulated genes such as *Ctnnb1* and *Aurka* encoding C‐Nap1‐binding potential β‐Catenin and Aurka are encountered, which may account for defective type B spermatogonia differentiation and meiotic entry in C‐Nap1‐null testes.

## 1 INTRODUCTION

In mammals, spermatogenesis commences during postnatal life and persists throughout an individual’s lifetime with continuous production from the postnatal day, and the consistency of spermatogenesis is greatly dependent on the existence of spermatogonia stem cells (SSCs). SSCs reside along the basal compartments of the testicular seminiferous epithelium. Over time, these SSCs demonstrate their capacity to differentiate into early spermatogonia with limited proliferation while also maintaining the SSC pool. Additionally, some early spermatogonia form cytoplasmic bridges through a series of mitotic divisions, which ultimately lead them toward differentiation into mature spermatogonia [1]. A‐type single spermatogonia, which is found along the basal compartments of testicular seminiferous epithelium, initiates mitotic divisions to produce either A paired spermatogonia or A aligned spermatogonia, where multiple spermatogonia are connected by intercellular bridges. Spermatogonia B, also known as type B spermatogonia, rises from A aligned. The process that spermatogonia develops from type A spermatogonia to B is called the differentiating process of spermatogonia. In addition, spermatogonia B will later develop into preleptotene spermatocytes, which will ultimately give rise to mature sperm cells [2].

The mammalian centrosome is the primary microtubule‐organizing center (MTOC) composed of two centrioles and the surrounding pericentriolar material (PCM) [3]. The two centrioles within a G1 centrosome are connected through a proteinous linker, emanating from their proximal ends. As cells progress from G1 into G2 phase, the centrosomes are duplicated, and the duplicated centrosomes remain linked to function as a single MTOC [4]. At the onset of mitosis, the linker is disassembled by Nek2A kinase‐mediated phosphorylation, coincident with centrosome separation in preparation for mitotic spindle assembly [5]. The centrosome linker C‐Nap1 localizing at the proximal ends of the centrioles acts as a docking site for all linker proteins [6]. The absence of *C‐Nap1* in non‐transformed RPE1 cells, also known as hTERT‐immortalized retinal pigment epithelial cells, results in a complete loss of centrosome linker activity [7]. It is shown this loss only leads to minor disruptions in cell migration and Golgi organization [8].

Recently, two studies report that *C‐Nap1* knockout (KO) male mice are infertile, but their phenotypic characterization in some aspects is contradictory. Moreover, the molecular mechanisms underlying the defective spermatogenesis of *C‐Nap1* KO mice remain poorly explored [9, 10]. Here, we show that the inactivation of C‐Nap1 in mice leads to defective spermatogenesis, rendering males infertile. Practically, we profile 10‐day testis samples from wild‐type (WT) and *C‐Nap1* KO mice using single‐cell RNA sequencing (scRNA‐seq) that enables the evaluation of gene expression at the single‐cell level and offers unprecedented insight into cellular heterogeneity. We identified the differentially expressed genes (DEGs) in spermatocytes and type B spermatogonia and evaluated their associated pathways through gene ontology (GO) enrichment analysis. We found that the C‐Nap1 negatively affects the transition from mitosis to meiosis during spermatogenesis in mouse testes.

## 2 RESULTS

### 2.1 C‐Nap1 KO impedes spermatogonia differentiation and meiotic entry

**FIGURE 1:**
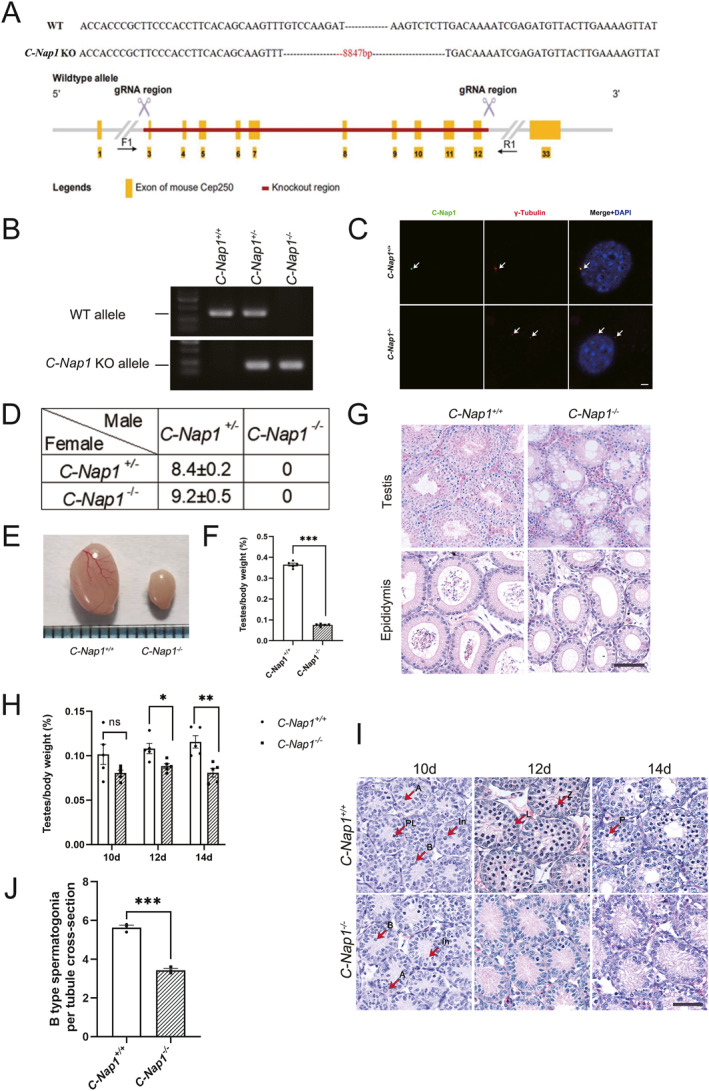
*C‐Nap1* null mice show defect spermatogenesis in the testes. (A) DNA sequencing analysis of *C‐Nap1* site deletion in KO mice and *C‐Nap1* genome knockout strategy and target design. (B) Genotypic identification of *C‐Nap1* KO mice. (C) IF was performed on both *C‐Nap1* ^*+/+*^ (WT) and *C‐Nap1* ^*−/−*^ MEFs. C‐Nap1 (green) and γ‐tubulin (red) markers were used, and DAPI (blue) was used to stain nuclei. Scale bar: 3 μm. (D) Male and female mice with *C‐Nap1* ^*−/−*^ and *C‐Nap1* ^*+/−*^ were utilized to conduct fecundity statistics. Mean litter sizes ± SEM are demonstrated according to its genotypes. (E) Testicles of 2‐month *C‐Nap1* ^*+/+*^ and *C‐Nap1* ^*−/−*^ mice. (F) Ratios of testis to body weight of *C‐Nap1* ^*+/+*^ and *C‐Nap1* ^*−/−*^ mice are demonstrated in (E) (*n* represents five individual experiments). Data are presented as mean ± SEM. (G) The stained sections of the *C‐Nap1* ^*+/+*^ and *C‐Nap1* ^*−/−*^ adult testes and epididymis, utilized hematoxylin/eosin stain. Scale bar: 100 μm. (H) Ratios of testis to body weight of *C‐Nap1* ^*+/+*^ and *C‐Nap1* ^*−/−*^ mice of 10, 12, and 14 dpp. (*n* represents five individual experiments). Data are presented as mean ± SEM. (I) Hematoxylin/eosin stain was used to stain sections of the *C‐Nap1* ^*+/+*^ and *C‐Nap1* ^*−/−*^ testes of 10, 12, and 14 dpp. Scale bar: 50 μm. (J) The stained testes sections from the *C‐Nap1* ^*+/+*^ and *C‐Nap1* ^*−/−*^ at 10 dpp with B type spermatogonia number per tubule. At least 50 tubules of each mouse were counted. (*n* represents three individual experiments). Data are presented as mean ± SEM. IF, Immunofluorescence; KO, knockout; MEFs, mouse embryonic fibroblasts; WT, wild‐type.

By using the CRISPR‐Cas9 system, *C‐Nap1* KO mice were constructed through deletion of exons 3–12 and the intervening introns of *C‐Nap1* (Figure 1A) were genotyped by genomic DNA PCR (Figure 1B). Absence of *C‐Nap1* expression in *C‐Nap1* KO mice was confirmed using immunofluorescence (IF). As shown in Figure 1C, C‐Nap1 was co‐localized with the centrosomal marker, γ‐tubulin at the centrosome of the mouse embryonic fibroblasts (MEFs) isolated from the *C‐Nap1* ^*+/+*^ mouse, but not from *C‐Nap1* ^*−/−*^. Of note, two widely separated γ‐tubulin‐positive dots were evident in *C‐Nap1* ^*−/−*^ MEFs.

After fecundity analysis of *C‐Nap1* mice, we observed that *C‐Nap1* ^*−/−*^ male mice produced zero offspring. On the other hand, *C‐Nap1* ^*−/−*^ female mice delivered normal litter size (Figure 1D). By comparing mouse testes, *C‐Nap1* ^*−/−*^ demonstrated an 80% reduction in the ratio of testis weight to body weight than its control litter mates (Figure 1E,F).

The histology of adult *C‐Nap1* ^*−/−*^ mouse revealed visible appearance of vacuole in the seminiferous tubules and no spermatozoon was identified in the epididymis. On the contrary, the histology of adult WT mice demonstrated no vacuole in the seminiferous tubules and many spermatozoa being present in the epididymis (Figure 1G). We thus conclude that *C‐Nap1* depletion contributes directly to azoospermia. Through measuring the testes to body weight of the two groups across postnatal days, we found that 12‐day testes started to show statistically significant differences, but no statistical difference prior to day 12 (Figure 1H). Testicular histology revealed that *C‐Nap1* ^*−/−*^ demonstrated less spermatocytes and spermatogonia compared to the WT mouse on postnatal 10 days (Figure 1I,J); furthermore, on 12 and 14 days, fewer spermatocytes and spermatogonia were demonstrated compared to WT (Figure 1I).

### 2.2 ScRNA‐seq analyses reveal spermatocyte loss in C‐Nap1‐null testes

**FIGURE 2:**
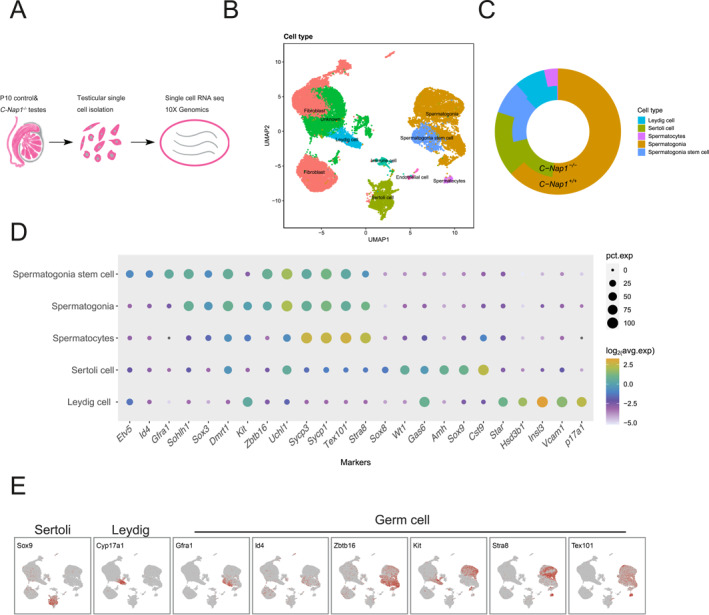
Overview of the primary cell categories and cellular characteristics obtained from single‐cell RNA sequencing of mouse *C‐Nap1* knocked out and WT. (A) A schematic flowchart illustrating this study. Firstly, the postnatal 10‐day mouse testis were selected as the targeted single‐cell analysis sample. Afterward, both postnatal 10‐day *C‐Nap1* ^*−/−*^ and WT testes samples were isolated for subsequent single‐cell analysis. Finally, 10× Genomics was applied to both WT and *C‐Nap1* ^*−/−*^ testes samples to obtain the output. (B) A visualization of UMAP clustering analysis of the single‐cell transcriptome data from P10 *C‐Nap1* ^*−/−*^ and WT testicular cells. A total of nine primary cell types, distinguished with individual color along with the name of cell types, were identified and presented on the UMAP plot using unsupervised clustering of 23,640 testicular cells. Each dot on UMAP represents a single cell, and cell clusters are visually highlighted by distinct colors corresponding to their respective cell types. (C) Layered ring chart of *C‐Nap1* ^*−/−*^ and WT, indicating their proportion of each testicular cell type. (D) A dot plot employed to visualize the expression of selected marker genes across five identified cell types (spermatogonia, Sertoli cell, spermatogonia stem cell, Leydig cell, and spermatocytes). Each cell type and marker gene are indicated with percentage expression and average logarithmic values. (E) Biological characterization of UMAP clustering analysis. The gene expression patterns of selected marker genes (*Sox9, Cyp17a1, Gfra1, Id4, Zbtb16, Kit, Stra8,* and *Tex101*) are depicted on the UMAP plot, where expression patterns of the nine selected marker genes on primary cell types showed gene localization. UMAP, uniform manifold approximation and projection; WT, wild‐type.

To explore the mechanisms underlying the early stage of defective spermatogenesis, we conducted scRNA‐seq to profile 10‐day testes of both WT and *C‐Nap1* KO mice (Figure 2A). A total of 11,114 *C‐Nap1* ^*+/+*^ and 13,902 *C‐Nap1* ^*−/−*^ testicular cells, 10,332 and 13,308 cells passed quality control (Figure S1). An observation of 10,880 UMIs (unique molecular indices) and 3526 genes in individual cells were detected. The identified data provided a suitable dataset to distinguish each cell type in mouse testis.

A total of 23,640 testicular cells obtained through unsupervised clustering were applied onto uniform manifold approximation and projection (UMAP) analysis, and nine major cell types for both *C‐Nap1* ^*+/+*^ and *C‐Nap1* ^*−/−*^ mice were identified (Figure 2B and Table S1). Based on each cluster’s expression patterns of known marker genes, individual identity was assigned to Leydig cells (*Cyp17a1, Vcam1,* and *Insl3*), Sertoli cells (*Cst9, Sox9,* and *Gas6*), spermatocytes (*Stra8, Tex101,* and *Sycp1*), spermatogonia (*Uchl1, Kit, Dmrt1,* and *Sox3*), and spermatogonia stem cells (*Gfra1, Id4,* and *Etv5*) (Figure 2D,E). We observed that the proportions of the main cell types in the seminiferous tubules are as follows: spermatogonia, Sertoli cells, spermatogonia stem cells, Leydig cells, and spermatocytes. In the *C‐Nap1* ^*+/+*^ mice, proportions were demonstrated to be 63.3%, 16.7%, 8.5%, 7.9%, and 3.5%, respectively. As for *C‐Nap1* ^*−/−*^ mice, the proportions were 51.9%, 19.3%, 16.5%, 11.8%, and 0.2% (Figure 2C). The ratio of spermatogonia was significantly reduced, which was consistent with the results observed in the tissue section (Figure 1I,J). Moreover, there were almost no spermatocytes in the testicles of *C‐Nap1* ^*−/−*^ mice. Altogether, these data suggest that spermatocytes are severely depleted in *C‐Nap1* ^*−/−*^ testes.

### 2.3 Single‐cell regulatory network inference and clustering

**FIGURE 3:**
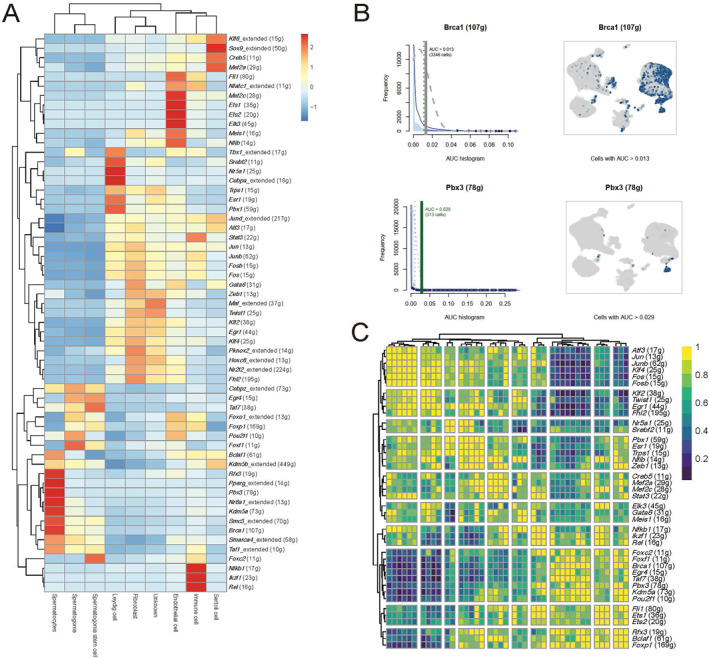
SCENIC transcriptome analysis. (A) A hierarchical clustering profile illustrates the expression levels of various gene markers across nine cell types, obtained through unsupervised clustering of 23,640 testicular cells. It highlights co‐regulated gene markers and cell types that exhibit similar communication patterns. The color scale represents the expression level of each gene marker across cell types, while “g” represents quantity. (B) AUC histograms are combined with spatial expression maps. The histograms depict the distribution of AUC values for *Brca1* and *Pbx3* across cells. Specifically, for *Brca1*, AUC >0.013 in 3346 cells, while for *Pbx3*, AUC >0.029 in 313 cells. The spatial expression maps display the locations of *Brca1* and *Pbx3* on the UMAP plot. (C) Multi‐parameter heat map visualizes the gene expression levels, functional annotations, and gene characteristics. AUC, area under the curve; SCENIC, single‐cell regulatory network inference and clustering; UMAP, uniform manifold approximation and projection.

In order to explore the intracellular communication patterns between different cell types within the testes and regulatory factors associated with the development of various cells, SCENIC (Single‐Cell Regulatory Network Inference and Clustering) was employed as the computational analysis method to demonstrate the regulatory factors such as Sertoli cell (*Kif6, Sox9, Creb5 and Mef2a*), Leydig cell (*Tbx1, Srebf2, Nr5a1, Cebpa, Esr1 and Pbx1*), spermatogonia stem cell (*Taf7, Egr4 and Foxc2*), spermatogonia (*Cebpz and Foxf1*), and spermatocytes (*Rfx3, Pparg, Pbx3, Nr6a1, Kdm5a, Smc3 and Brca1*) (Figure 3A) [11]. Among these, there are certain regulatory factors associated with sperm development in spermatocytes. For example, *Brca1* (Figure 3B), a DNA‐damage repair and crossing‐over gene, has been shown to play an essential role in spermatogenesis [12, 13]. Similarly, *Pbx3*, another specific regulator of spermatocytes, is considered an important transcription factor in mouse spermatogenesis [14]. Furthermore, a multi‐parameter heat map was demonstrated to visualize the gene expression level along with functional annotations and characteristics (Figure 3C). Taken together, these data suggest a detailed analysis of the molecular mechanisms underlying testicular cell development and function, with a focus on the identification of key regulatory genes and their interactions.

### 2.4 Transcriptome signatures of C‐Nap1^−/−^ testes

**FIGURE 4:**
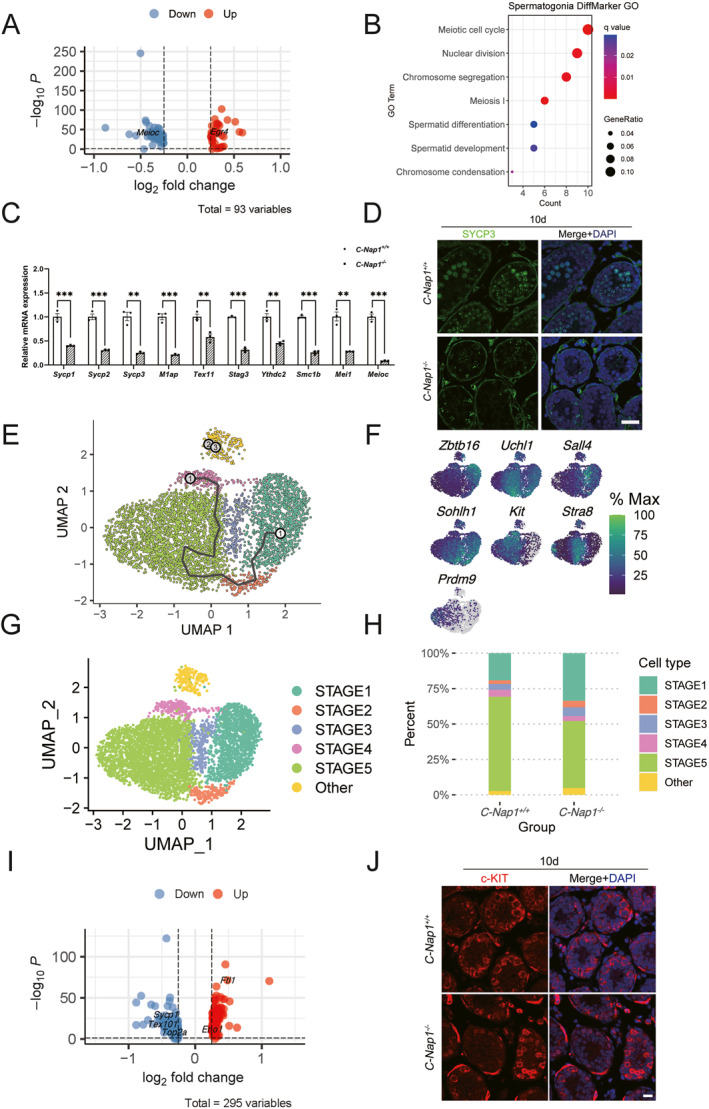
The deficiency of *C‐Nap1* ^*−/−*^ alters the expression pattern of spermatogenesis in mice. (A) The volcano plot illustrates the single‐cell transcriptome of *C‐Nap1* ^*−/−*^ and WT testis at postnatal day 10. *Meioc* is shown to be downregulated, whereas *Egr4* is demonstrated to be upregulated. (B) Gene ontology term versus spermatogonia differential marker graph represented with gene ratio and *q*‐values. (C) The mRNA expression of meiosis‐specific genes in *C‐Nap1* ^*+/+*^ and *C‐Nap1* ^*−/−*^ testes at P10 was measured by quantitative RT‐PCR. GAPDH was used as the internal control. (D) Immunofluorescence analysis of the meiotic specific marker SYCP3 (green) in 10 days testis sections. DNA was stained with DAPI. Scale bar: 30 μm. (E) In a focused analysis (combining *C‐Nap1* ^*−/−*^ and WT P10 testis cells with pseudotime ordering and UMAP clustering), three cellular states were identified in chronological spermatogenesis development, encompassing five spermatogonial states from STAGE1 to STAGE5. (F) Gene expression patterns of seven selected marker genes corresponding to each spermatogonial state on the UMAP, differentiated by gene expression intensity. (G) UMAPs depict the combined spermatogonial development states in *C‐Nap1* ^*−/−*^ and WT P10 testes cells. A total of five states were identified including an unknown state. (H) The bar chart displays the proportion of each spermatogonial development state in *C‐Nap1* ^*−/−*^and WT P10 testes cells. (I) The volcano plot illustrates downregulated and upregulated genes in STAGE5. (J) Immunofluorescence analysis of the differentiated spermatogonia where cell marker c‐KIT (red) in 10 days testis sections were applied. DNA was stained with DAPI. Scale bar: 20 μm. RT‐PCR, reverse transcription polymerase chain reaction; UMAP, uniform manifold approximation and projection; WT, wild‐type.

While spermatocytes are the cell types with significant differences in cell proportions in the testis, we also observed differences between *C‐Nap1* ^*+/+*^ and *C‐Nap1* ^*−/−*^ in earlier spermatogonia. Subsequently, we employed differential gene expression (DEG) analysis on the spermatogonia cells of *C‐Nap1* ^*+/+*^ and *C‐Nap1* ^*−/−*^, and 93 DEGs were identified (*P*_adj_ < 0.05, Figure 4A and Table S2). GO analysis revealed that these DEGs are enriched in functional terms related to meiotic processes, including meiotic cell cycle, nuclear division, chromosome segregation, meiosis I, spermatid differentiation, and chromosome condensation (Figure 4B and Table S2). Following the foregoing analyses, we performed the RT‐PCR in 10‐day *C‐Nap1* ^*+/+*^ and *C‐Nap1* ^*−/−*^ testes, showing a number of meiosis‐specific genes greatly down‐regulated in KO group (Figure 4C), ascertained by immunostained with an antibody against the meiotic‐specific marker SYCP3 (Figure 4D).

To pinpoint the specific spermatogenesis stage affected by *C‐Nap1*, we re‐clustered 2806 *C‐Nap1* ^*+/+*^ and 2391 *C‐Nap1* ^*−/−*^ spermatogonia cells. To identify the cell types, marker genes analysis and UMAP were employed to assist analysis. By combining the results of expression distribution of marker genes, UMAP, and monocles3 [15] (Figure 4F), we identified five distinct spermatogonia subtypes/states (STAGE1 to STAGE5) (Figure 4G). In *C‐Nap1* ^*+/+*^ samples, we determined that 19.2% were in STAGE1, 2.5% in STAGE2, 4.1% in STAGE3, 4.9% in STAGE4, 66.5% in STAGE5, and 2.7% in others (Figure 4H). In *C‐Nap1* ^*−/−*^ samples, 33.6% were in STAGE1, 4.6% in STAGE2, 6.2% in STAGE3, 3.4% in STAGE4, 47.3% in STAGE5, and 4.8% in others. A drastic drop in the *C‐Nap1* ^*−/−*^ samples of STAGE 4 and 5 stayed consistent with the decrease in spermatogonia as a small proportion of testicular cells. DEG analysis between *C‐Nap1* ^*+/+*^ and *C‐Nap1* ^*−/−*^ cells in STAGE5 subtypes revealed that *Sycp1*, *Tex101*, and *Top2a*, known to play important roles in spermatogenesis, were significantly decreased in mutant STAGE5 cells (Figure 4I).

According to the results of pseudotime series analysis and the expression pattern of late spermatogonial markers (Figure 4E,F), STAGE5 appears to correspond to type B spermatogonial cells, and the proportion of STAGE5 cells in the testes of KO mice was significantly reduced (Figure 4H). As IF demonstrated, the differentiated spermatogonia marked by c‐KIT expression were significantly less in KO seminiferous tubules than that in the WT (Figure 4J), confirming the decrease in type B spermatogonia. Therefore, we believe *C‐Nap1* deficiency affects the development of late spermatogonia.

### 2.5 High‐dimensional weighted correlation network analysis

**FIGURE 5:**
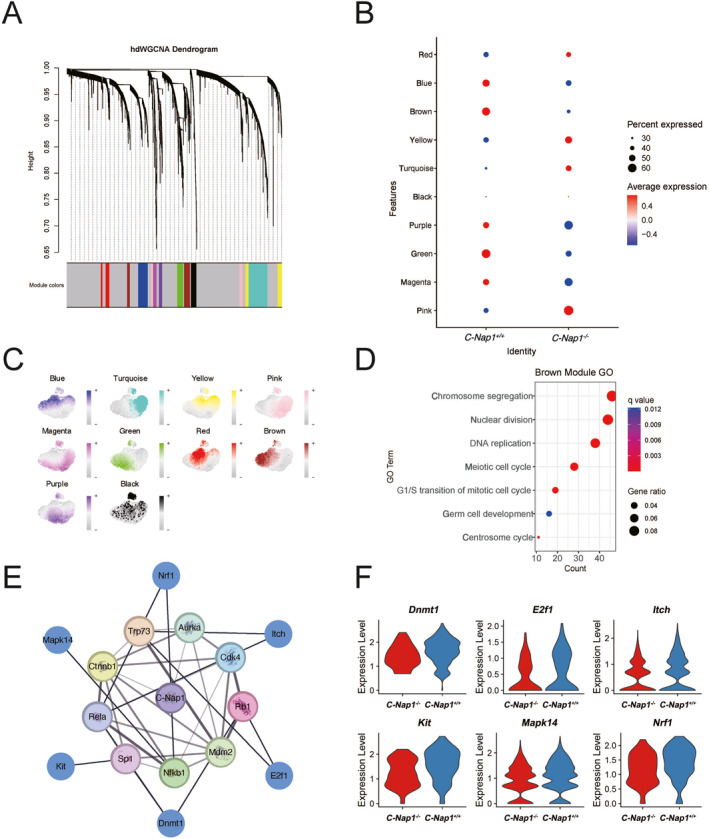
High‐dimensional weighted gene co‐expression network analysis (hdWGCNA). (A) Dendrogram along with its network modules is demonstrated. Gray modules were discarded and not utilized in the subsequent analysis. (B) Dot plots illustrate the module‐specific gene expression levels based on module colors. Marker genes and their expression levels for both WT and *C‐Nap1* ^*−/−*^ are displayed. (C) The clustering tree displays the relationships between the modules. (D) Gene ontology terms with brown module markers are represented by gene ratios and *q*‐values. (E) A protein network illustrates protein–protein associations with C‐Nap1 at the center and its neighboring proteins, demonstrating their interactions with C‐Nap1. (F) Violin plots display the number of expression levels for a total of six types of gene markers, including *Dnmt1, E2f1, Itch, Kit, Mapk14,* and *Nrf1*. WT, wild‐type.

To explore the genes associated with *C‐Nap1* on spermatogenesis, we employed high‐dimensional weighted gene co‐expression network analysis (hdWGCNA) [16] for further analysis on spermatogonia. Based on the scale‐free network structure, we selected 14 (as the soft‐thresholding power) to identify co‐expression networks and we obtained a total of 10 modules (Figure 5A). After assessing the expression levels in each module across different genotypes and expressed cell types, we concluded that the brown module demonstrated a high association with the reduced cell population of spermatogonia (Figure 5B,C). Through GO enrichment analysis in the brown module (between green and black), these DEGs were primarily enriched in functional terms related to spermatogenesis (Figure 5D). After comparing the modules, we hypothesized that the brown module corresponds to STAGE5 as depicted in Figure 4G. Therefore, we used the intersection of DEGs in STAGE5 and genes in the brown module as the genes representing the phenotypic effects in KO mice (Table S4).

To understand how *C‐Nap1* affects the expression levels of these genes, we used the TRRUST database [17] to predict the transcription factors for these genes, and we used the STRING database [18] to predict protein interaction networks related to C‐Nap1, followed by selecting DEGs associated with these proteins in STAGE5 (Figure 5E). These DEGs all showed significant down‐regulation (Figure 5F). We speculate that this down‐regulation may have contributed to the loss of *C‐Nap1*, consequently affecting spermatogenesis.

## 3 DISCUSSION

During steady‐state spermatogenesis, several crucial transitions occur, including from undifferentiated spermatogonia to type A1 differentiating spermatogonia and from type B differentiating spermatogonia to preleptotene spermatocytes that are prepared for the initiation of meiosis [1, 19].

In this study, we affirmed that *C‐Nap1* is fundamental for spermatogenesis, and the absence of *C‐Nap1* would result in male infertility. Recently, research has reported the phenotype of *C‐Nap1*‐deficient mice [10], which demonstrated similar results to our study. All research has found that the lack of *C‐Nap1* causes centrosome splitting (Figure 1C), and one of the reports suggests that the premature separation of centrosomes activates the arrest of meiosis. For additional investigations, we take advantage of scRNA‐seq to unravel the heterogeneity and complexity of RNA transcripts within individual cells of 10‐day WT and *C‐Nap1* KO testes. We analyzed nine major cell types and found that Sertoli cells and Leydig cells showed no significant changes (Figure 2C; Table S1), indicating that *C‐Nap1* KO does not interfere with the development of testicular somatic cells. Such finding aligns with the previous report [10]. It is worthy to note that cell clustering indicated a drastic reduction in spermatogonia and spermatocytes; in addition, C‐Kit staining also confirmed the reduction of differentiated spermatogonia (Figure 4J), while spermatogonia stem cells are not affected. This finding is consistent with the previous report. Moreover, the researchers believed that *C‐Nap1* affects the late differentiation of spermatogonia but were uncertain about the specific time and cell type. Another report suggests that *C‐Nap1* deficiency affects the differentiation of germ stem cells, mainly causing premature differentiation of spermatogonia, which leads to defects in spermatogonia in maintaining germ stem cells [10]. However, our experimental results show that the early spermatogonia are not affected (Figures 2C and 4H). To determine at which specific stage sperm cell development was affected, additional clustering analysis of spermatogonia, pseudotime analysis, and cell proportion analysis were performed (Figure 4E,H). We identified a significant difference that occurred in the late stage of spermatogonial development (STAGE5). Since B‐type spermatogonia represent the final stage of mitotic division in spermatogonia, the reduced cells in the KO mouse testes are most likely B‐type spermatogonia (Figures 1J and 4J). Thus, *C‐Nap1* KO affects spermatogonial differentiation and subsequent mitosis‐to‐meiosis germ cell transition, the two processes that are controlled by the transcription of suites of key regulatory genes [20].

Based on the DEGs in STAGE5, we predicted some upstream transcription factors that may be related to these DEGs, by which β‐catenin encoded by *Ctnnb1* and Aurka may potentially have a direct interaction with C‐Nap1. Two possible scenarios are thus proposed. First, β‐catenin is a well‐studied protein with known functions in the nucleus in a complex with the transcriptional coactivator T‐cell factor/lymphocyte enhancer factor‐1 (TCF/Lef‐1) [21]. β‐Catenin also partially colocalizes with C‐Nap1 and is in a complex with the other known centrosomal linker Rootletin. Depletion of *C‐Nap1* causes the loss of Rootletin and β‐catenin from the intercentrosomal linker region [22], probably resulting in the downregulation of these DEGs. Whether this downregulation is the cause of the reduction in B‐type spermatogonia remains unclear and requires further investigation. Another possibility is that disruptions in the self‐activation process of aurora kinase A (AURKA) affect spermatogenesis. Aurka locates in centrosomes and spindle fibers, playing a crucial role in centrosome maturation, separation, entry into mitosis, and assembly of bipolar spindle fibers. Loss of *Aurka* in germ cells has been reported to lead to reproductive cell development failure [23]. Therefore, we speculate that *C‐Nap1* deficiency may impact the expression of *Aurka*, thereby affecting the development of late‐stage spermatogonia.

## 4 CONCLUSION

In summary, our study demonstrated the centrosomal protein C‐Nap1 to be an essential component for male mouse spermatogenesis, and C‐Nap1 is required for the transition of spermatogonia from mitosis to meiosis. The absence of *C‐Nap1* would result in a reduction in B‐type spermatogonia, but the mechanisms underlying *C‐Nap1* regulation in this context require further investigation. In addition, we mined the protein network related to C‐Nap1 through single cell sequencing and screened out some genes related to meiosis during spermatogenesis, hoping to provide some help for the study of male reproduction.

## 5 MATERIALS AND METHODS

### 5.1 Animals

Cyagen Biosciences constructed the KO mice of *C‐Nap1* by using the CRISPR‐Cas9 system. The genotyping primers for KO were F1: 5′‐ ACCCACTCCCGCAGACAGAAACT‐3′, R1: 5′‐AACTCACACTGAGCCATCTTGACA‐3′, and for WT mice, the primers were F2: 5′‐ TAGTTATTGAGCATCAGGGACAC‐3′, R1: 5′‐AACTCACACTGAGCCATCTTGACA‐3′. Mice were kept in the same animal house and underwent a simulated environment, which was repeated day and night (12 h each). Approval for animal experiments and protocols were obtained and granted by the Institutional Animal Care and Use Committee (IACUC) of the Institute of Zoology, Chinese Academy of Sciences, China (IOZ20180012).

### 5.2 Antibodies

Wuhan Dai'an Biotechnology Co., ltd generated the antibody. Rabbit polyclonal anti‐C‐Nap1 was used in this study. Other antibodies that were also employed in this study are listed in Table S3.

### 5.3 Fertility assay

To evaluate mice fertility, in a 6‐month time spend, we caged five *C‐Nap1* ^*−/−*^ and *C‐Nap1* ^*+/−*^ female mice together with either *C‐Nap1* ^*+/−*^ or *C‐Nap1* ^*−/−*^ male mice, and we quantified the average number of pups per litter.

### 5.4 MEFs

*C‐Nap1* ^*+/−*^ male and female mice were combined in cages and plugged female mice were retrieved after 12.5 days. The pregnant mice were killed by dislocation and the embryos were removed immediately by laparotomy. After the embryo was stripped of viscera, head, and limbs in PBS, the chopped tissues were added with 3 mL pancreatic enzyme, digested in a 37°C incubator for 5 min, and then the same amount of DMEM medium was added, after blowing and mixing, and then 7 mL medium was added, and cultured in a 37°C incubator. The cell status was observed the next day and was replaced with a fresh medium.

### 5.5 Histology and IF

Testes dissected from WT and KO mice immediately after Anle’s death were immobilized overnight in 4% paraformaldehyde or Bouin solution, dehydrated with graded concentrations of ethanol, treated with xylene, and then paraffin‐embedded. 5 μm thick slices were cut and affixed to the slides. After xylene de‐waxing and gradient concentration ethanol rehydration, Bouin‐fixed testicle sections were used for HE staining and PFA‐fixed testicle sections were used for IF analysis.

After dewaxing, rehydration, and antigenic repair with sodium citrate buffer, slides were washed three times using PBS, which contained 5% bovine serum albumin to block for 50 min. The initial antibody was applied to the slices and allowed to incubate at 4°C for a minimum of 12 h. Subsequently, a secondary antibody was used at a 1:200 dilution and incubated for 1 h at 37°C. Nuclei were counterstained with DAPI.

MEFs cells were grown on cover slips, rinsed in PBS, and fixed in −20°C methanol for 8 min. The cells were washed with PBS three times and then permeated with 0.2% Triton X‐100 for 5 min, rinsed in PBS three times, and blocked in 4% bovine serum albumin in PBS for 40 min. Then, incubated with primary antibodies at 4°C for more than 12 h, and incubated with secondary antibody at 1:200 for 50 min at room. The nuclei were stained with DAPI.

### 5.6 Quantitative real‐time PCR

Total RNA was extracted from whole testes using TRIGene reagent (GenStar, P118‐05) following the manufacturer’s instructions. Subsequently, 1 μg of total RNA was reverse transcribed into cDNAs with Hifair® II 1st Strand cDNA Synthesis Kit (YeaSen, 11121ES60*) according to the manufacturer’s protocol. Quantitative real‐time PCR was carried out using Hieff UNICON® qPCR SYBR® Green Master Mix (YeaSen, 11198ES08) in a CFX96 Touch Real‐Time PCR Detection System (Bio‐Rad). Relative mRNA expression was calculated based on the 2^−ΔΔCt^ method with GAPDH as an internal control. Three biological replicates were performed for each sample. All primers are listed in Table S5.

### 5.7 Statistical analysis

Data presented in this study utilized mean ± standard error of mean. A two‐tailed Student’s *t*‐test with paired two‐tails distribution was performed to obtain the statistical significance of differences between genotypes' mean values using SPSS and GraphPad Prism 8. The data were considered significant when * *p*‐value <0.05, ** *p*‐value <0.01, and *** *p*‐value <0.0001. *n* = 5.

### 5.8 Single‐cell sequencing analysis

Testicular single‐cell isolation was conducted on both the P10 controlled group and P10 *C‐Nap1* ^*−/−*^ samples. Single‐cell RNA‐seq using 10× Genomics Cell Ranger 7.2.0 [24] was employed following its standard workflow (including demultiplexing, alignment, gene expression quantification, quality control, clustering and analysis, and output) to generate the single‐cell dataset for subsequent analysis. R 4.2.1, provided by the R Foundation for Statistical Computing 2022 (R core team 2022), was used as the programming environment with the Seurat software package version 4.4.0 (version 4.1.1, [25]) for data processing, which included pre‐processing, dimensionality reduction, clustering, DEG analysis, and trajectory analysis as well as for visualizations and statistical analysis.

The single‐cell samples from 10× Genomics Cell Ranger 7.2.0 were filtered based on RNA count (200 < RNA count <7388) and mitochondrial genes cut off was set to be less than 20% of the total dataset. SCTransform v2 function 0.4.1 (version 0.3.3, [26]) was used for batch correction, z‐score calculation, and variability modeling to normalize the dataset. For cell quantification, CellMarker 2.0 (version 2.0, [27]) was employed to identify marker genes in cell populations (as an outcome of the processing).

To explore the transcriptome‐wide signature of spermatogonia stem cell (SSC) development and the associated clusters, we integrated the dataset to identify cell groups based on marker gene expression. This analysis revealed clusters including fibroblast, unknown, Leydig cell, immune cell, Sertoli cell, endothelial cell, spermatogonia, SSC, and spermatocytes (Figure 2B). Differential expression analysis was performed with a log fold change threshold (logfc.threshold) of 0.25 and a minimum percentage required for a particular criterion (min.pct) of 0.1.

For cell–cell communication analysis, Nichenet 2.0.4 (version 2.0.4, github website (saeyslab/nichenetr), Cambridge, UK, accessed on 16 December 2023 [28]) and CellPhonedb 4.1.0 (version 2.0.4, github website (ventolab/CellphoneDB), Ghent, Belgium, accessed on 16 December 2023 [29]) were used to quantify cellular populations and their functional interactions.

SCENIC was utilized for transcriptome analysis (version 1.3.1, github website (aertslab/SCENIC), Leuven, Belgium, accessed on 16 December 2023 [11]) and TRRUST (version 2, [17]). WGCNA analysis was performed using hdWGCNA (version 0.2.24, [16]).

RNA velocity analysis utilized scvelo (version 0.2.5, github website (theislab/scvelo), Munich, Germany, accessed on 16 December 2023 [30]).

Pseudotime analysis was conducted using Monocle3 (version 1.3.4, [31]), which provided a chronological developmental analysis with an arrow vector indicating the order of SSCs from spermatogonial STAGE1 to 5 (Figure 4E). STRINGdb (STRING database in 2023, Zurich, Switzerland, accessed on 16 December 2023 [32]) was used for protein–protein interaction analysis with C‐Nap1 at the center to visualize its interactions with nearby proteins based on relatedness (encoded with *C‐Nap1*) (Figure 5E).

## AUTHOR CONTRIBUTIONS

**Junlin Li**: Methodology; writing – original draft; writing – review & editing. **Liheng Yang**: Investigation; software; writing – original draft. **Liansheng Li**: Methodology. **Min Li**: Methodology. **Juntao Gao**: Conceptualization; supervision; writing – review & editing. **Li Yuan**: Conceptualization; supervision; writing – review & editing.

## CONFLICT OF INTEREST STATEMENT

The authors Junlin Li, Liheng Yang, Liansheng Li, Min Li, Li Bao, Juntao Gao, and Li Yuan declare that they have no conflict of interest or financial conflicts to disclose.

## ETHICS STATEMENT

All procedures performed in studies involving animals were in accordance with the ethical standards of the institution or practice at which the studies were conducted.

## Supporting information

Table S1

Table S2

Table S3

Table S4

Table S4

Figure S1

## Data Availability

The data that support the findings of this study are open source and available from the corresponding website mentioned in the article.
