# Supplementary material for: Single‐cell analyses reveal impaired type B spermatogonia differentiation and meiotic entry in C‐Nap1‐null testes
Source: Quant Biol. 2024 Nov 26;13(1):e71. doi: 10.1002/qub2.71 (PMC12806081; doi:10.1002/qub2.71)
Supplement: Supplementary file 3 — Table S3 [file QUB2-13-e71-s006.docx]

Table S3. List of antibodies.

| **Antibody** | **Host** | **Company** | **Dilution** | **Cat#** |
| --- | --- | --- | --- | --- |
| **c-KIT** | **Rabbit** | **Cell Signaling** | **IF: 1:200** | **D13A2** |
| **SYCP3** | **Rabbit** | **made by our laboratory** | **IF: 1:500** |  |
| **C-Nap1** | **Rabbit** | **Wuhan Dai 'an Biotechnology Co., LTD** | **IF: 1:5000** | **S-2326-1** |
| **γ-Tubulin** | **Mouse** | **Santa Cruz** | **IF: 1:2000** | **sc51715** |
| **Alexa Fluor 594** **anti-rabbit** | **Goat** | **Invitrogen** | **IF: 1:1500** | **A11007** |
| **Alexa Fluor 488 anti-mouse** | **Goat** | **Invitrogen** | **IF: 1:1500** | **A11006** |
