# Supplementary material for: Single‐cell analyses reveal impaired type B spermatogonia differentiation and meiotic entry in C‐Nap1‐null testes
Source: Quant Biol. 2024 Nov 26;13(1):e71. doi: 10.1002/qub2.71 (PMC12806081; doi:10.1002/qub2.71)
Supplement: Supplementary file 5 — Table S4 [file QUB2-13-e71-s004.docx]

Table S5 **Primers for quantitative real-time PCR**

| Gene | Forward (5'-3') | Reverse (5'-3') |
| --- | --- | --- |
| *Sycp1* | CAAAAGCCCTTCACACTGTTCG | GTTTTCCCGACTGGACATTGTAA |
| *Sycp2* | AGGATGAGATCACTACACCTAGC | GGTGACGCAGCATAATCCATT |
| *Sycp3* | AGCCAGTAACCAGAAAATTGAGC | CCACTGCTGCAACACATTCATA |
| *M1ap* | CAACCTCCGAGGCTTCTCATT | CATGCTGGTTCTGTACTGTGTAT |
| *Tex11* | ATGAAGCTCCCTCAACTTTGG | CCCTCTTTTGCACTTTGTGGA |
| *Stag3* | GGACCATGTCTTTCTCCAGCC | TAGAGCTGCTTTAGGCTCAGG |
| *Ythdc2* | GGTCCGATCAATCATCTGT | GAAGTAACGAATAGGCATGT |
| *Smc1b* | ACACAGTTTTCGGCCTGCTC | CCAGAGTCAACACATGGCTG |
| *Mei1* | CAGGCTGTTCACAAGCTCAG | GAAGTGCTCTGTACTGCACG |
| *Meioc* | CTGAGGGAGGGACCTGAGCC | ATCATAGAAGGGCGCCGAGC |
