# Supplementary material for: Single‐cell analyses reveal impaired type B spermatogonia differentiation and meiotic entry in C‐Nap1‐null testes
Source: Quant Biol. 2024 Nov 26;13(1):e71. doi: 10.1002/qub2.71 (PMC12806081; doi:10.1002/qub2.71)
Supplement: Supplementary file 6 — Figure S1 [file QUB2-13-e71-s001.docx]

**
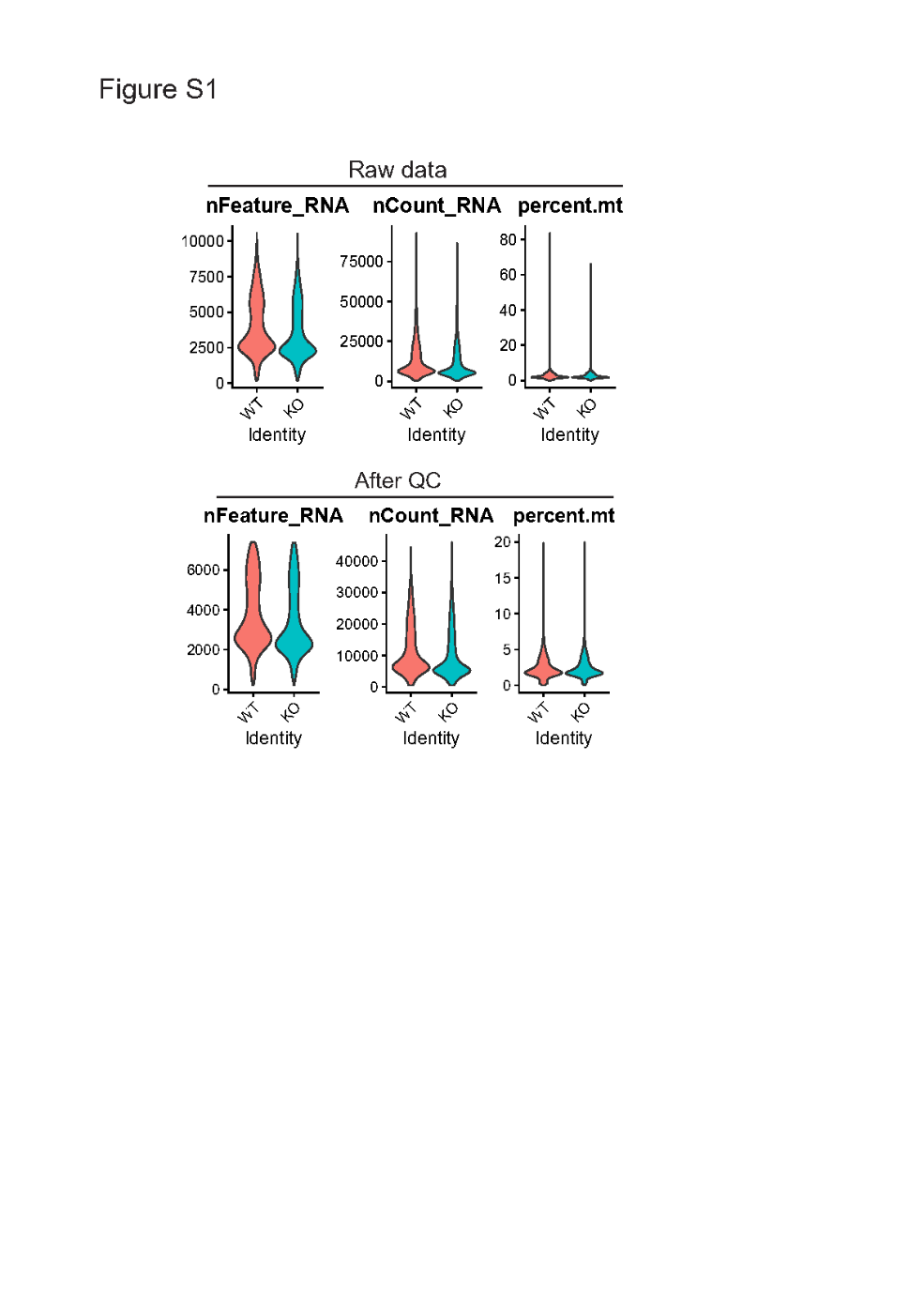
**

Figure S1. Single-cell RNA-seq of P10 *C-Nap1^+/+^* (WT) and *C-Nap1^-/-^* (KO) testicular cells by 10×Genomics. Quality control (QC) metrics of raw and post-QC data for *C-Nap1^+/+^* and *C-Nap1^-/-^*. n = 11,114 for *C-Nap1^+/+^* and n = 13,902 for *C-Nap1^-/-^* in raw data; n =10,332 for *C-Nap1^+/+^* and n =13,308 for *C-Nap1^-/-^* in post-QC data.
